# Country of origin and prices of systemic antibiotics in Vietnam: a multicentre retrospective study

**DOI:** 10.1093/jacamr/dlae221

**Published:** 2025-01-16

**Authors:** Vu Quoc Dat

**Affiliations:** Department of Infectious Diseases, Hanoi Medical University, No 1 Ton That Tung Street, Dong Da District, Hanoi, Vietnam; Hanoi Medical University Hospital, Hanoi Medical University, No 1 Ton That Tung Street, Dong Da District, Hanoi, Vietnam

## Abstract

**Background:**

Local production of antibiotics is essential for improving access to treatment of clinical infection and avoiding vulnerability to expensive drug imports.

**Objectives:**

To describe the country of origin and cost of antibiotics in Vietnam.

**Methods:**

This was an observational study. Antibiotic procurement in 372 healthcare facilities in Vietnam between 2018 and 2022 were included in this analysis. Antibiotics were classified using the Anatomical Therapeutic Chemical Index and the 2021 WHO Access, Watch and Reserve groups. The country of origin of antibiotics was determined by the place where the final products were manufactured. Antibiotic costs were estimated in US dollars per DDD and adjusted by annual inflation.

**Results:**

This study included 2.54 billion antibiotic DDDs, totalling 3.13 billion US dollars. Local production accounted for 80.2% of the number of DDDs and 43.4% of the total expenditure. The antibiotics from other countries were driven by imports from high-income countries, which accounted for 75.5% of DDDs and 89.6% of the expenditure on imported antibiotics. Availability of Reserve group antibiotics was more dependent on imports from high-income countries (36.6% of DDDs) than those of Access (15.7% of DDDs) and Watch group antibiotics (14.2% of DDDs).

**Conclusions:**

A comprehensive policy approach is needed to ensure the affordability and to reduce dependency on imported Reserve group antibiotics.

## Introduction

Global antibiotic consumption increased by 46% between 2000 and 2018, reaching 14.3 DDDs per 1000 inhabitants per day.^1^ In 2020, spending on antibiotics and vaccines in high- and upper-middle-income country markets totalled US $35–37 billion (ranking sixth for spending on medicine), and totalled $20–21 billion in emerging pharmaceutical markets (ranking third for spending on medicine).^2^

Vietnam is one of 22 countries that are defined as ‘pharmerging’ markets, i.e. countries with per capita gross domestic product (GDP) less than $30 000/year and forecasted 5 year aggregate pharma sales growth greater than $1 billion.^3^ The value of the Vietnam pharmaceutical market reached US $4.2 billion in 2014 and substantially increased to US $5.62 billion in 2018 and is forecast to reach 12.6 billion in 2025.^4^ In 2021, Vietnam had 222 local manufacturers that were certified with WHO Good Manufacturing Practices (GMP) standards and 18 manufacturers that were certified with EMA GMP standards.^5^ The overall value of domestic medicine used by the healthcare facilities increased from 46.6% in 2013 to 63.3% in 2018.^6^ The favourable domestic production of medicine in Vietnam explained the sharp increase in the country’s domestic drug consumption during the past 10 years. A country-level goal of using domestic medicine for at least 80% of all medicine usage in healthcare insurance is included in the national strategy by 2030.^5^

Vietnam was the country with the highest increase in antibiotic usage in Southeast Asia, with an increase of 15% from 2000 to 2018.^1^ In a setting with a high prevalence of antibiotic resistance (e.g. at a high prevalence of 52% of patients having colonization with carbapenem-resistant Enterobacteriaceae, as reported in a point prevalence survey of 12 Vietnamese hospitals^7^), when an average daily cost of antibiotics to treat carbapenem-resistant Gram-negative bacteria was US $172,^8^ which costs almost 17 times the daily income in Vietnam (GDP per capita of US $3551^9^), it is clearly not generally affordable. When medicine costs account for 52.7% of health expenditure,^5^ it becomes a challenge for national budgets. If antibiotics are produced locally, it is expected that their price would be cheaper than imported products. However, new antibiotic substances for treatment of MDR pathogens are often first introduced in high-income countries (HICs), initially in the USA and European countries, and spread through other lower- to middle-income countries (LMICs) 5 to 10 years after the first market introduction.^10^ Attention to the balance of costs and volume of antibiotics between imports and local production is crucial to achieving affordability and improving the availability and access to essential antibiotics. The aim of this study was to describe the market shares and the prices of available antibiotics, by the manufacturers’ country of origin, in Vietnam.

## Materials and methods

### Ethics

This study did not involve human subjects and did not require institutional review board (IRB) approval.

### Study setting

Vietnam is an LMIC in Southeast Asia with an estimated population of 100.4 million people as of 2023.^11^ The health sector in Vietnam is divided into public and private sectors, in which the public sector has 34 national hospitals, 417 provincial hospitals, 1034 district hospitals/clinics and 755 hospitals/health centres that operate under different government ministries, serving specific populations (hereinafter all referred to as the healthcare facilities) and together accounting for 96% of the total number of hospital beds nationwide.^12^ The bidding process is a method used to procure drugs. In public drug procurement, the procurement units can be the national centralized procurement units (assigned by the Ministry of Health), the local centralized procurement units (assigned by the provincial People’s Committee) or individual healthcare facilities to undertake procurement for medicines.^13^

### Data sources

The search was performed on the website of the Drug Administration of Vietnam, Ministry of Health, and the antibiotic consumption was estimated using the data of successful bid results from public healthcare facilities that were publicly available between 2018 and 2022 as national policy required for medicine procurement. Antibiotics for systemic use (J01) defined by the Anatomical Therapeutic Chemical Classification System (ATC) were selected for analysis.^14^ The AWaRe (Access, Watch and Reserve) classification of antibiotics was used to describe the antibiotic usage patterns. The data extraction included details about procured antibiotics, including active ingredients, route of administration, country of origin, pharmaceutical manufacturers, quantity of purchase, expenditure, and levels of healthcare facilities. The country of origin was defined by where a final production (finished dosage form) was manufactured and it was classified as a low-, lower middle-, upper middle- or high-income by the World Bank country classifications in 2022.^15^

### Variable definition

DDD is defined as the average dose per day for a drug used for an adult of 70 kg for its main indication. It was used to standardize the comparison of antibiotic procurement by countries of origin or the comparison of prices between different drugs. The expenditure per DDD was calculated for the actual cost paid for specific antibiotic substances or AWaRe antibiotic groups, which allowed comparison of international differences in the expenditure for the same drug. DDDs are not established for all medicines with an ATC code, therefore it was not possible to calculate the cost per DDD; this study excluded systemic antibiotics without DDD-assigned factors from the final analysis. The price of each item was weighted by its number of DDDs. All expenditure was adjusted for inflation to the 2022 US dollar value using the consumer price index (CPI) and the annual average official exchange rates: US $1 = 22 602.05 Vietnamese dong (VND) in 2018 and 23 271.21 VND in 2022.^16^

The expense ratio was calculated by dividing the percentage of expenditure by the percentage of DDDs in the same antibiotic class by its origin. The higher the expense ratio, the higher the spending an antibiotic procurement was relative to its country of origin. The price ratio of local production to imports was defined as the ratio between the quantity weighted price per DDD of domestic antibiotic to that of imported antibiotics for the same antibiotic substance or AWaRe antibiotic group.

### Statistics

Statistical analysis was performed in IBM SPSS Statistics for Windows (version 27.0, IBM Corp., Armonk, NY, USA), with *P* < 0.05 considered to be statistically significant.

## Results

This analysis included the successful bid results of 2.54 billion DDDs for antibiotics, totalling over 3.13 billion US dollars from 372 procurement units in Vietnam between 2018 and 2022. There was a total of 76 antibiotic substances (including 22 in the Access group, 44 in the Watch group, 7 in the Reserve group and 4 in the non-recommended group) (fosfomycin was in both groups of Watch and Reserve) that were supplied by 88 domestic and 247 foreign manufacturers (Table 1). Among countries and territories exporting antibiotics to Vietnam, 22/36 (66.1%) were high-income regions (with 135 manufacturers), 8/36 (22.2%) were upper-income regions (with 24 manufacturers) and 6 (16.7%) were lower middle-income regions (with 88 manufacturers). India was the country with the highest numbers of foreign manufacturers (63/247; 25.5%), followed by South Korea (31/247; 12.6%) and Spain and Germany (each with 31/247; 5.7%).

**Table 1.** Characteristics of antibiotic supply in the healthcare facilities

Characteristics of Vietnam pharmaceutical market	All	Antibiotics originating from Vietnam	Antibiotics originating from other countries
Total antibiotic expenditure, $ (%)	3 130 123 841	$1 358 643 101 (43.4)	$1 771 480 741 (56.6)
Total number of DDDs, *n* (%)	2 544 435 681	2 041 890 275 (80.2)	502 545 407 (19.8)
Numbers of manufacturers, *n* (%)	335	88 (26.3)	247 (73.7)
Numbers of available antibiotics, *n* (%)			
Access group antibiotics	25	24/25 (96.0)	19/25 (76.0)
Watch groups antibiotics	45	45/45 (100.0)	41/45 (91.1)
Reserve group antibiotic	7	3/7 (42.9)	7/7 (100.0)
Not recommended^a^	10	10/10 (100.0)	5/10 (50.0)
Proportion of antibiotic purchase by AWaRe classification, DDDs (%)			
Access group antibiotics	1 113 088 445	868 573 774 (78.0)	244 514 671 (22.0)
Watch groups antibiotics	1 418 810 836	1 165 552 840 (82.1)	253 257 997 (17.9)
Reserve group antibiotic	2 794 657	1 437 650 (51.4)	1 357 006 (48.6)
Not recommended^a^	9 741 743	6 326 010 (64.9)	3 415 733 (35.1)
Proportion of antibiotic purchase by level of procurement units, DDDs			
Centralized bidding (*n* = 65)	2 249 700 717	1 823 033 031 (89.3)	426 667 686 (84.9)
District hospitals (*n* = 124)	51 967 461	47 093 883 (2.3)	4 873 578 (1.0)
Provincial hospitals (*n* = 148)	205 753 355	154 938 376 (7.6)	50 814 979 (10.1)
National hospitals (*n* = 38)	37 014 149	16 824 985 (0.8)	20 189 164 (4.0)

^a^Not-recommended antibiotics included fixed-dose combinations of multiple broad-spectrum antibiotics whose use is not recommended by the WHO AWaRe classification due to the lack of evidence-based indications.

Our pooled data, which aggregated centralized and decentralized procurement units, show that during the 5 year period, the Vietnam antibiotic manufacturing sector made up 80.2% (range 73.5%–83%) of the total number of DDDs and 43.4% (range 35.8%–57.4%) of the total expenditure of the antibiotics market, while the remaining 19.8% (range 17%–26.5%) of systemic antibiotics were imported from 36 different countries/territories and accounted for 56.6% (range 42.6%–64.2%) of total antibiotic expenditure (Table 1). Among imported antibiotics, HICs accounted for 75.5% of DDDs (379.5 million DDDs), totalling 89.6% (US $1.59 billion) of the expenditure, compared with just 21.3% of DDD and 6.9% (US $122.8 million) of the expenditure in LMICs. The remaining small proportions of imported antibiotics were from upper middle-income countries (UMICs) (3.2% or 15.9 million DDD and 3.4% or US $60.4 million of expenditure). The overall expense ratios were 3.4 for manufacturers from HICs, 3.1 for those from UMICs, 0.9 for those from LMICs and 0.5 for domestic manufacturers in Vietnam.

**Figure 1.**
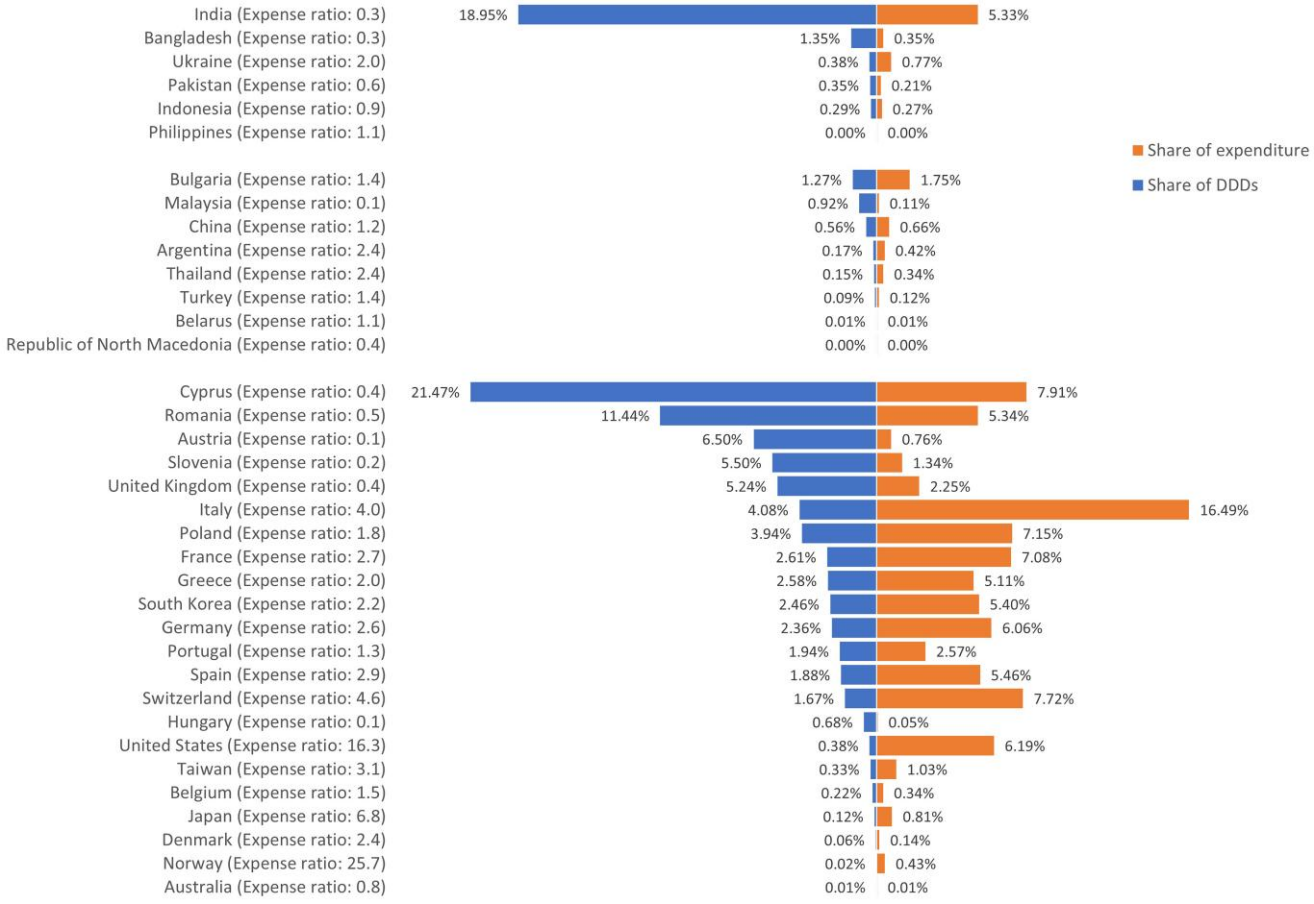
List of sources of antibiotic imports in percentage of DDD numbers and expenditure.

**Figure 2.**
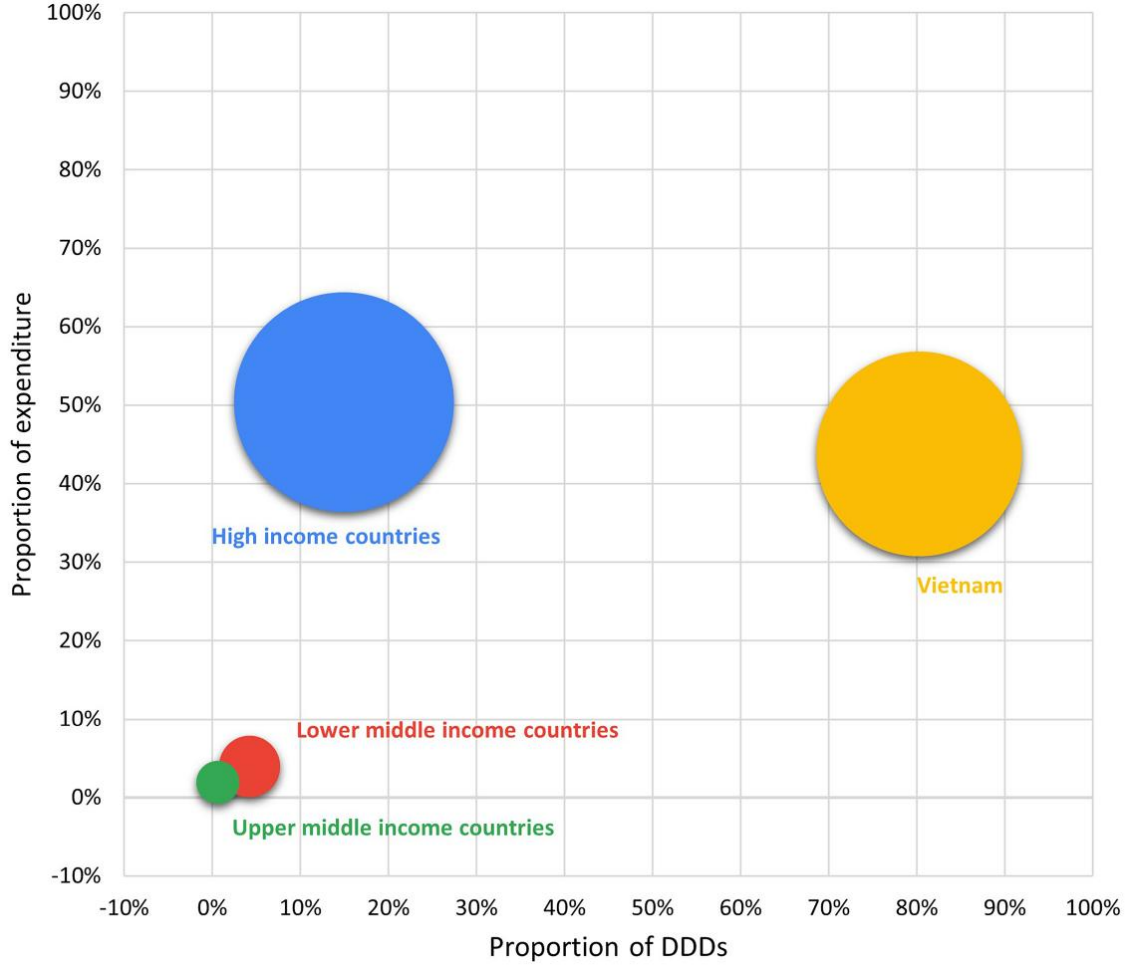
Bubble diagram showing the shares of expenditure (*y*-axis) and proportions of DDDs (*x*-axis) by country income level. Size (area) of bubbles represents the number of DDDs.

Cyprus, Bulgaria and India were the largest exporters of antibiotics from HICs, UMICs and LMICs in terms of number of DDDs, accounting for 28.4% (107.9 million), 40% (6.4 million) and 88.8% (95.2 million) of DDDs in the corresponding income country levels, respectively. A list of the sources of antibiotic imports is shown in Figure 1. The largest volumes of antibiotic imports from any single country came from Cyprus (21.5%), India (19%) and Romania (11.4%), nearly double to three times the amount of the next largest source (on a DDD proportion basis), which together accounted for 51.9% (260.6 million) of total imported antibiotics but only 18.6% (US $329.3 million) of total budget for imported antibiotics (Figure 2).

**Figure 3.**
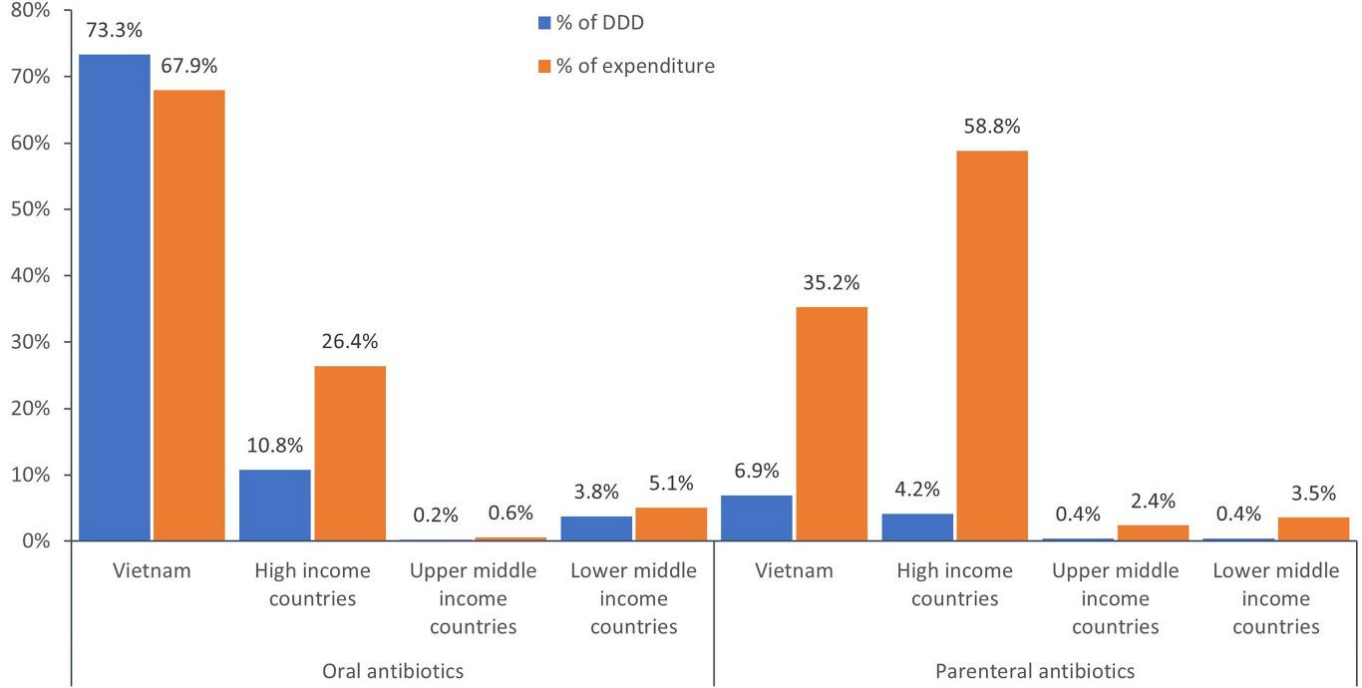
The share of antibiotic markets by country of origin and route of administration.

Overall, oral antibiotics accounted for 88.1% (2.25 billion DDDs) and parenteral antibiotics for 11.9% (303.4 million DDDs) of the total DDDs, corresponding to 25% (US $780.9 million) and 75% (US $2.35 billion) of the total expenditure, respectively. HICs accounted for only 12.2% (273.7 million) of oral antibiotic DDDs but 34.9% (105.8 million) of parenteral antibiotic DDDs, while Vietnam accounted for 83.2% (1.87 billion) of oral antibiotics and 58.2% (176.7 million) of parenteral antibiotic DDDs. The remaining quantities of antibiotics were shared by UMICs and LMICs (4.6% or 102.2 billion DDDs of oral antibiotics, and 6.9% or 20.9 million DDDs of parenteral antibiotics). The share of quantities and expenditure of antibiotics by route of administration is shown in Figure 3. The antibiotic consumption and antibiotic expenditure over years by the antibiotic country of origin are shown in Tables S1 and S2 (available as Supplementary data at *JAC-AMR* Online).

Antibiotic supply between domestic and foreign manufacturers by AWaRe classification is shown in Table 1. Domestic manufacturers supplied 78% (868.6 million DDDs) of Access group antibiotics and 82.1% (1.17 billion DDDs) of Watch group antibiotics, while manufacturers from HICs supplied 15.7% (175 million DDDs) and 14.2% (201.5 million DDDs) of Access and Watch group antibiotics, respectively. However, the proportion of Reserve group antibiotics supplied by HICs increased to 36.6% (1 million DDDs), in which Japan accounted for the largest share of imported Reserve group antibiotics from HICs (30.9% or 316 100 DDDs), followed by Poland (29.9% or 305 855 DDDs) and Spain (9% or 91 502 DDDs). The percentage of procured antibiotic imports by country of origin is shown in Figure S1. Four out of five of completely imported antibiotics (daptomycin, tigecycline, ceftazidime/β-lactamase inhibitor, ceftolozane/β-lactamase inhibitor) were Reserve group antibiotics, which accounts for four out of seven available antibiotics in this group. The three remaining Reserve group antibiotics of linezolid (50.8%, 1.4 million DDDs), colistin (24.95%, 695 170 DDDs) and fosfomycin (21%, 587 947 DDDs) were still the most commonly used Reserve group antibiotics and together accounted for 96.7% of Reserve group antibiotics (in DDDs), with the percentage of imports being 43.7% (619 634 DDDs), 40.6% (282 008 DDDs) and 61.6% (362 168 DDDs), respectively.

The volume-weighted average price per DDD was $0.69 for Access group antibiotics, $1.45 for Watch group antibiotics, and $53.56 per DDD for Reserve group antibiotics (Table 2 and Table S3). The top three most expensive antibiotics were Reserve group antibiotics: ceftazidime/β-lactamase inhibitor ($357.45 per DDD); ceftolozane/β-lactamase inhibitor ($216.18 per DDD); and colistin ($150.84 per DDD).

**Table 2.** Prices of antibiotics by AWaRe classification and country of origin

Antibiotics	Route of administration	HICs	UMICs	LMICs	Vietnam
Access group					
Aminoglycosides	Parenteral	$3.79 (6.63)	$2.97 (5.20)		$0.57
Amphenicols	Oral				$0.27
	Parenteral				$1.49
β-Lactam/β-lactamase inhibitor	Oral	$0.94 (1.93)	$2.33 (4.82)	$0.49 (1.01)	$0.48
	Parenteral	$11.66 (2.17)	$3.87 (0.72)	$9.68 (1.80)	$5.39
β-Lactamase inhibitors	Parenteral	$18.49 (0.00)			
First-generation cephalosporins	Oral	$0.67 (1.92)	$0.25 (0.71)	$0.95 (2.73)	$0.35
	Parenteral	$6.35 (1.24)	$2.99 (0.58)	$4.88 (0.95)	$5.14
Imidazoles	Parenteral	$4.28 (3.03)	$2.97 (2.10)	$1.40 (0.99)	$1.41
Lincosamides	Oral	$2.17 (5.23)			$0.41
	Parenteral	$14.63 (3.94)			$3.72
Penicillins	Oral	$0.38 (2.15)		$0.18 (1.03)	$0.18
	Parenteral	$6.00 (1.72)			$3.48
Tetracyclines	Oral	$0.07 (3.87)		$0.02 (1.18)	$0.02
Watch group					
Aminoglycosides	Parenteral	$6.39 (2.54)		$6.39 (2.54)	$2.51
β-Lactam/β-lactamase inhibitors, anti-pseudomonals	Parenteral	$19.12 (1.12)		$12.50 (0.73)	$17.07
Carbapenems	Parenteral	$38.24 (2.02)	$84.41 (4.46)	$29.15 (1.54)	$18.92
Fluoroquinolones	Oral	$0.74 (7.33)	$0.57 (5.64)	$0.13 (1.30)	$0.10
	Parenteral	$10.42 (2.30)	$6.59 (1.46)	$6.34 (1.40)	$4.52
Fourth-generation cephalosporins	Parenteral	$14.33 (2.22)		$22.68 (3.51)	$6.46
Glycopeptides	Parenteral	$12.81 (2.51)	$9.76 (1.91)	$7.67 (1.50)	$5.10
Lincosamides	Oral				$0.21
Macrolides	Oral	$1.28 (8.32)	$1.34 (8.71)	$0.70 (4.56)	$0.15
	Parenteral	$12.83 (3.47)		$3.79 (1.02)	$3.70
Penicillins	Parenteral	$39.70 (1.79)			$22.13
Phosphonics	Oral	$5.42 (1.19)			$4.54
Second-generation cephalosporins	Oral	$0.73 (2.89)	$0.56 (2.22)	$0.38 (1.51)	$0.25
	Parenteral	$14.89 (1.88)	$7.82 (0.99)	$2.59 (0.33)	$7.93
Tetracyclines	Oral	$2.45 (1.95)			$1.26
Third-generation cephalosporins	Oral	$1.63 (3.76)	$2.53 (5.85)	$1.13 (2.61)	$0.43
	Parenteral	$5.99 (2.08)	$6.74 (2.34)	$1.66 (0.58)	$2.88
Reserve group					
Fifth-generation cephalosporins	Parenteral	$216.18 (0.00)			
Glycylcyclines	Parenteral	$68.51 (0.00)		$62.95 (0.00)	
Lipopeptides	Parenteral			$41.72 (0.00)	
Oxazolidinones	Oral			$1.61 (1.20)	$1.34
	Parenteral	$66.82 (3.45)		$25.22 (1.30)	$19.37
Phosphonics	Parenteral	$37.38 (1.97)		$21.53 (1.14)	$18.93
Polymyxins	Parenteral	$150.84 (1.29)		$102.67 (0.88)	$116.87
Third-generation cephalosporins	Parenteral	$357.45 (0.00)			

The numbers in brackets refer to the ratio of the price of imported drugs from HICs, UICs and LMICs to the price of domestic drugs in Vietnam.

The average prices of imported antibiotics from HICs were not lower than those produced domestically, regardless of being oral or parenteral antibiotics. Among the same antibiotics being produced domestically or imported from other LMICs, the average price of imported antibiotics was higher than those of locally produced antibiotics in 19/21 oral antibiotics (11/19 antibiotics with price ratios between 1 and 2; 6/19 antibiotics with price ratio between 2 and 5; and 2/19 antibiotics with price ratios above 5) and in 18/26 parenteral antibiotics (15/18 antibiotics with price ratios between 1 and 2; and 3/18 antibiotics with price ratios between 2 and 3). The details of antibiotic prices are shown in Table 2.

## Discussion

This was the largest study reporting the 5 year market shares and prices of antibiotics in Vietnam. The vast majority of the antibiotics consumed in Vietnam were domestically produced, although dependence on the imports of Reserve group antibiotics was higher than those of Access and Watch group antibiotics. There was a disproportionate distribution between budget shares and quantity of antibiotics by local production and imports, in which the domestic manufacturers accounted for 80.2% of total antibiotic consumption (in DDDs) but foreign manufacturers accounted for 56.6% of the total expenditure.

National surveillance data published by the Vietnam Ministry of Health in 2023 showed that among 69 715 bacterial isolates from clinical specimens in 16 hospitals, the prevalence of carbapenem-resistant *Escherichia coli* (CREC) was about 10%, carbapenem-resistant *Klebsiella pneumoniae* (CRKP) was about 50%, MRSA was 78%, vancomycin-resistant *Enterococcus faecium* (VREfm) was 26.2%, and penicillin-resistant *Streptococcus pneumoniae* was 91.5% (using the susceptibility breakpoint for meningeal infections) and 15.2% (using the susceptibility breakpoint for non-meningeal infections).^17^ Under the high burden of antibiotic resistance, it is important to improve appropriate access to Reserve antibiotics to address the morbidity and mortality caused by resistant pathogens. There are currently no reports on the clinical outcomes associated with the improvement of access to Reserve antibiotics in Vietnam. However, at the regional level (Southeast Asia, East Asia and Oceania), 18.74 million deaths could be cumulatively averted between 2025 and 2050, through better care and improved access to antibiotics.^18^

There are five main levels of the pharmaceutical value chain.^19^ Level 1 is the import of finished pharmaceutical products (FPPs). Level 2 is the packaging and labelling of already formulated products. Level 3 is the formulation of finished dosage products from imported active pharmaceutical ingredients (APIs) and excipients. Level 4 is production of active pharmaceutical ingredients and excipients, and level 5 is the active research and development for new formulations and new chemical entities. By 2022, the majority of the players in Vietnam pharmaceutical sectors were at level 3, in which the domestic manufacturers accounted for only 5.2% for APIs and 9.8% for excipients in term of monetary value.^12^

According to the Observatory of Economic Complexity (OEC), in 2021 Vietnam ranked as the world’s 13th highest importer of antibiotics, with the total trade of US $246 624 255, which accounted for 2.37% of the total global trade of antibiotics (US $10.4 billion).^20^ The top exporters of antibiotics to Vietnam were China (US $175 million, 71%), India (US $45.7 million, 18.5%), Spain (US $10.6 million, 4.29%) and Italy ($2.89%, 1.17%).^20^ These estimates relied on the raw data from the United Nations Statistical Division and Base pour l’Analyse du Commerce International (BACI).^21,22^

In 2021, the Vietnam’s spending on healthcare was US $173 per capita, while out-of-pocket spending for health was 40%.^9^ The national demand for pharmaceutical products is growing rapidly as economies grow but the local pharmaceutical industry has faced competitive disadvantages. These have included regulatory obstacles (such as issues related to registration, certification, licensing and the procurement process), technical barriers to developing new, safe and effective innovative drugs, and barriers to sustainability in pharmaceutical supply chains. In 2021, the 74th World Health Assembly called for strengthening of local production of medicines and other health technologies to improve access. It urged countries to develop policies supporting sustainable local pharmaceutical production, to promote research and development, to increase transparency of markets for medicines, to enhance collaboration, and to form partnerships to build and improve the transfer of technology.^23^ In 2024, the WHO announced six successful country case studies in LMICs (Bangladesh, Kenya, Nigeria, Pakistan, Senegal and Tunisia), as lessons for other countries.^24–29^

Generic essential medicines can be produced at a very low price, in comparison with those under patent protection or brand name medicines.^30^ The case studies of India and Brazil in the past 20 years have demonstrated that local production can produce competitive assured-quality medicines.^31^ However, there are several barriers to local production of medicines in LMICs, such as limited human resources, poor infrastructure, lack of collaborative technology transfer and poor adherence to GMP standards.^32^ The current situation is that despite the low price of locally produced antibiotics, Vietnam has had an expenditure leakage into imports of antibiotics in all categories of the AWaRe classification. In November 2023, Vietnam’s prime minister released Vietnam’s national strategy for developing the pharmaceutical industry through to 2030, with a vision for 2045 of ensuring affordable access to medications for the public. Investment incentive policies for research and technology transfers, production of innovative drugs, pharmaceutical raw materials and medication originating from Vietnamese medicinal herbs and limiting the import of pharmaceutical raw materials that Vietnam has produced are among key solutions.^33^ The government should adopt the WHO recommendation for further strengthening the framework to support local production and improve affordability of last-resort antibiotics for the treatment of MDR and XDR pathogens.^32,34^

Antibiotic shortage was considered to be a global health concern before the emergence of SARS-CoV-2 in 2019 and became more alarming during the COVID-19 pandemic between 2020 and 2023. Before the COVID-19 pandemic, the antibiotic shortage was related to the limited availability of low-cost, useful traditional antibiotics due to the low profit margin, the supply chain issues and the dependency on a few manufacturers for pharmaceutical raw materials in China and India^35,36^ and to the limited accessibility of new antibiotics due to the insufficient profitability.^37^ In a study of antibiotic sales in 71 countries (including Vietnam) between 2020 and 2022, a 10% increase in monthly COVID-19 cases contributed to higher sales growth of cephalosporins by 0.2%–0.3%, penicillins by 0.2%–0.3%, and macrolides by 0.4%–0.6%.^38^ In a systematic review of 74 studies from January 2000 to July 2023, the frequent causes of antibiotic shortages were identified as disruption in manufacturing (including lack of supply of APIs and excipients), the lack of financial incentive to produce low-cost antibiotics and demand and supply–demand imbalances.^39^

The main limitation of this study was the classification of a country of origin of an antibiotic, based on the place where a final product was manufactured rather than where an API was produced. This can lead to an underestimate of the value of imports because the majority of APIs needed to be imported for the final products by local manufacturers in Vietnam, and the price of APIs, generally accounted for the most significant component of the cost of medicines. Another limitation was the convenience sampling method that did not allow evaluation of the changes in balance of local production and imports over time because the datasets of antibiotic procurement were not obtained with the same procurement units for consecutive years during 2018–23. Furthermore, the study period included the COVID-19 pandemic period in Vietnam, in which the country could experience antibiotic shortages as a result of the changes in demand and global supply chain disruptions. However, the retrospective nature of this study, the convenience sampling method and limited data variables did not permit analysis of changes in antibiotic demand and pricing over time, especially for imported antibiotics.

In conclusion, the majority of expenditure on imports of antibiotic went to HICs. At the high prices of imported antibiotics and the dependence on manufactures from HICs for Reserve group antibiotics, Vietnam should consider allocating resources to local production and improvement of policies to increase the affordability and accessibility of essential Reserve group antibiotics.

## Supplementary Material

dlae221_Supplementary_Data

## Data Availability

The data that support the findings of this study are available from the corresponding author upon reasonable request.

## Supplementary data

Figure S1 and Tables S1–S3 are available as Supplementary data at *JAC-AMR* Online.
