# Supplementary material for: Country of origin and prices of systemic antibiotics in Vietnam: a multicentre retrospective study
Source: JAC Antimicrob Resist. 2025 Jan 16;7(1):dlae221. doi: 10.1093/jacamr/dlae221 (PMC11735462; doi:10.1093/jacamr/dlae221)
Supplement: dlae221_Supplementary_Data [file dlae221_supplementary_data.zip › Supplementary_tables.docx]

Supplementary table 1. The proportion of defined daily dose (DDDs) by country of origin between 2018 and 2022

|  | Other countries | | | | | Vietnam | | |  |  | Total |
| --- | --- | --- | --- | --- | --- | --- | --- | --- | --- | --- | --- |
| Antibiotic class | **2018** | **2019** | **2020** | **2021** | **2022** | **2018** | **2019** | **2020** | **2021** | **2022** |  |
| Reserve group antibiotics | **0.26%** | **0.19%** | **0.50%** | **0.32%** | **0.28%** | **0.06%** | **0.07%** | **0.11%** | **0.08%** | **0.09%** | **0.11%** |
| Oxazolidinones | 0.12% | 0.08% | 0.23% | 0.15% | 0.13% | 0.03% | 0.04% | 0.06% | 0.05% | 0.04% | 0.06% |
| Polymyxins | 0.04% | 0.06% | 0.10% | 0.08% | 0.06% | 0.01% | 0.02% | 0.03% | 0.03% | 0.04% | 0.03% |
| Phosphonics | 0.09% | 0.05% | 0.13% | 0.07% | 0.05% | 0.02% | 0.01% | 0.01% | 0.00% | 0.01% | 0.02% |
| Fifth-generation cephalosporins | 0.00% | 0.00% | 0.00% | 0.00% | 0.00% | 0.00% | 0.00% | 0.00% | 0.00% | 0.00% | 0.00% |
| Glycylcyclines | 0.01% | 0.01% | 0.02% | 0.01% | 0.02% | 0.00% | 0.00% | 0.00% | 0.00% | 0.00% | 0.00% |
| Lipopeptides | 0.00% | 0.00% | 0.00% | 0.01% | 0.01% | 0.00% | 0.00% | 0.00% | 0.00% | 0.00% | 0.00% |
| Third-generation-cephalosporins | 0.00% | 0.00% | 0.00% | 0.00% | 0.01% | 0.00% | 0.00% | 0.00% | 0.00% | 0.00% | 0.00% |
| Watch group antibiotics | **52.39%** | **50.89%** | **35.74%** | **49.22%** | **54.32%** | **59.09%** | **61.11%** | **48.19%** | **49.92%** | **59.97%** | **55.98%** |
| Second-generation-cephalosporins | 14.34% | 20.18% | 2.05% | 9.45% | 19.72% | 23.63% | 34.37% | 6.33% | 13.62% | 24.74% | 21.37% |
| Third-generation-cephalosporins | 10.28% | 11.21% | 7.24% | 9.06% | 7.24% | 14.93% | 13.28% | 20.54% | 16.06% | 15.67% | 14.20% |
| Fluoroquinolones | 15.32% | 12.13% | 17.86% | 14.65% | 19.88% | 9.75% | 6.10% | 8.64% | 8.17% | 11.34% | 10.14% |
| Macrolides | 9.61% | 2.94% | 5.37% | 12.45% | 1.08% | 10.35% | 6.59% | 11.81% | 11.24% | 6.88% | 8.95% |
| Carbapenems | 1.14% | 2.59% | 0.25% | 2.22% | 4.12% | 0.07% | 0.28% | 0.05% | 0.16% | 0.17% | 0.50% |
| Aminoglycosides | 0.60% | 0.55% | 1.02% | 0.18% | 0.22% | 0.17% | 0.19% | 0.41% | 0.26% | 0.59% | 0.30% |
| Fourth-generation-cephalosporins | 0.57% | 0.65% | 0.54% | 0.37% | 0.90% | 0.06% | 0.12% | 0.03% | 0.04% | 0.19% | 0.19% |
| Glycopeptides | 0.30% | 0.32% | 0.68% | 0.46% | 0.57% | 0.07% | 0.08% | 0.16% | 0.16% | 0.18% | 0.16% |
| Beta-lactam/beta-lactamase-inhibitor (anti-pseudomonal) | 0.13% | 0.19% | 0.35% | 0.21% | 0.48% | 0.03% | 0.06% | 0.12% | 0.12% | 0.14% | 0.10% |
| Penicillins | 0.04% | 0.08% | 0.27% | 0.12% | 0.06% | 0.02% | 0.04% | 0.08% | 0.05% | 0.04% | 0.05% |
| Phosphonics | 0.08% | 0.03% | 0.05% | 0.04% | 0.04% | 0.00% | 0.00% | 0.00% | 0.00% | 0.00% | 0.01% |
| Tetracyclines | 0.00% | 0.01% | 0.06% | 0.01% | 0.00% | 0.00% | 0.01% | 0.02% | 0.03% | 0.02% | 0.01% |
| Lincosamides | 0.00% | 0.00% | 0.00% | 0.00% | 0.00% | 0.00% | 0.00% | 0.00% | 0.00% | 0.00% | 0.00% |
| Access group antibiotics | **47.35%** | **48.92%** | **63.77%** | **50.46%** | **45.40%** | **40.85%** | **38.82%** | **51.70%** | **50.00%** | **39.94%** | **43.91%** |
| Beta-lactam/beta-lactamase-inhibitor | 18.54% | 22.51% | 15.14% | 14.05% | 27.13% | 13.51% | 16.77% | 11.21% | 16.72% | 9.76% | 15.23% |
| Penicillins | 17.36% | 19.12% | 32.09% | 22.65% | 9.27% | 13.23% | 9.83% | 20.51% | 17.21% | 12.74% | 14.60% |
| First-generation-cephalosporins | 7.26% | 5.39% | 11.65% | 6.43% | 5.07% | 10.60% | 10.53% | 17.08% | 12.86% | 15.20% | 10.95% |
| Tetracyclines | 1.50% | 0.69% | 1.63% | 3.74% | 1.63% | 2.13% | 0.58% | 0.63% | 1.12% | 1.01% | 1.45% |
| Aminoglycosides | 0.66% | 0.39% | 1.39% | 1.51% | 0.44% | 0.76% | 0.65% | 1.50% | 0.95% | 0.64% | 0.80% |
| Imidazoles | 1.48% | 0.70% | 1.66% | 1.69% | 1.66% | 0.42% | 0.35% | 0.63% | 0.92% | 0.50% | 0.68% |
| Lincosamides | 0.35% | 0.12% | 0.19% | 0.28% | 0.21% | 0.19% | 0.10% | 0.15% | 0.23% | 0.08% | 0.18% |
| Beta-lactamase-inhibitors | 0.20% | 0.00% | 0.03% | 0.12% | 0.00% | 0.00% | 0.00% | 0.00% | 0.00% | 0.00% | 0.02% |
| Amphenicols | 0.00% | 0.00% | 0.00% | 0.00% | 0.00% | 0.00% | 0.00% | 0.00% | 0.00% | 0.00% | 0.00% |
|  |  |  |  |  |  |  |  |  |  |  |  |

Note: Vietnam reported the first COVID-19 cases in January 2020 (the first wave of COVID-19 in April 2020) and transited to a 'new normal' during the COVID-19 pandemic in May 2022. Because of the potential bias due to the incomplete and non-representative nature of the dataset, the results of this table should be interpreted with caution

Supplementary table 2. The share of antibiotic expenditure by country of origin between 2018 and 2022

|  | 2018 | | 2019 | | 2020 | | 2021 | | 2022 | |
| --- | --- | --- | --- | --- | --- | --- | --- | --- | --- | --- |
| Antibiotic class | **Other countries** | **Vietnam** | **Other countries** | **Vietnam** | **Other countries** | **Vietnam** | **Other countries** | **Vietnam** | **Other countries** | **Vietnam** |
| Reserve group antibiotics | **62.34%** | **37.66%** | **57.10%** | **42.90%** | **61.95%** | **38.05%** | **53.33%** | **46.67%** | **65.22%** | **34.78%** |
| Fifth-generation cephalosporins |  |  |  |  | 100.00% | 0.00% | 100.00% | 0.00% | 100.00% | 0.00% |
| Glycylcyclines | 100.00% | 0.00% | 100.00% | 0.00% | 100.00% | 0.00% | 100.00% | 0.00% | 100.00% | 0.00% |
| Lipopeptides |  |  |  |  |  |  | 100.00% | 0.00% | 100.00% | 0.00% |
| Oxazolidinones | 81.33% | 18.67% | 78.03% | 21.97% | 73.85% | 26.15% | 73.11% | 26.89% | 84.81% | 15.19% |
| Phosphonics | 71.24% | 28.76% | 80.39% | 19.61% | 81.70% | 18.30% | 84.26% | 15.74% | 79.29% | 20.71% |
| Polymyxins | 49.28% | 50.72% | 47.21% | 52.79% | 49.79% | 50.21% | 40.93% | 59.07% | 45.31% | 54.69% |
| Third-generation-cephalosporins |  |  |  |  |  |  | 100.00% | 0.00% | 100.00% | 0.00% |
| Watch group antibiotics | **65.24%** | **34.76%** | **70.35%** | **29.65%** | **42.39%** | **57.61%** | **51.52%** | **48.48%** | **58.11%** | **41.89%** |
| Aminoglycosides | 64.20% | 35.80% | 63.63% | 36.37% | 51.83% | 48.17% | 26.43% | 73.57% | 42.42% | 57.58% |
| Beta-lactam/beta-lactamase-inhibitor (anti-pseudomonal) | 52.15% | 47.85% | 53.44% | 46.56% | 40.26% | 59.74% | 31.42% | 68.58% | 55.40% | 44.60% |
| Carbapenems | 88.79% | 11.21% | 89.22% | 10.78% | 42.89% | 57.11% | 81.49% | 18.51% | 91.98% | 8.02% |
| Fluoroquinolones | 81.15% | 18.85% | 79.58% | 20.42% | 68.79% | 31.21% | 65.29% | 34.71% | 83.15% | 16.85% |
| Fourth-generation-cephalosporins | 80.92% | 19.08% | 81.24% | 18.76% | 91.68% | 8.32% | 83.55% | 16.45% | 76.26% | 23.74% |
| Glycopeptides | 62.23% | 37.77% | 75.50% | 24.50% | 74.23% | 25.77% | 68.72% | 31.28% | 78.43% | 21.57% |
| Lincosamides | 0.00% | 100.00% |  |  |  |  |  |  |  |  |
| Macrolides | 61.80% | 38.20% | 47.74% | 52.26% | 44.38% | 55.62% | 62.87% | 37.13% | 38.17% | 61.83% |
| Penicillins | 44.64% | 55.36% | 48.00% | 52.00% | 55.86% | 44.14% | 45.46% | 54.54% | 43.59% | 56.41% |
| Phosphonics | 100.00% | 0.00% | 99.22% | 0.78% | 98.09% | 1.91% | 75.83% | 24.17% | 89.65% | 10.35% |
| Second-generation-cephalosporins | 49.89% | 50.11% | 58.50% | 41.50% | 15.04% | 84.96% | 33.68% | 66.32% | 31.47% | 68.53% |
| Tetracyclines | 0.00% | 100.00% | 29.90% | 70.10% | 54.69% | 45.31% | 7.52% | 92.48% | 8.46% | 91.54% |
| Third-generation-cephalosporins | 52.62% | 47.38% | 56.16% | 43.84% | 28.31% | 71.69% | 28.98% | 71.02% | 19.74% | 80.26% |
| Access group antibiotics | **44.20%** | **55.80%** | **36.53%** | **63.47%** | **38.20%** | **61.80%** | **35.80%** | **64.20%** | **44.94%** | **55.06%** |
| Aminoglycosides | 57.94% | 42.06% | 54.32% | 45.68% | 56.92% | 43.08% | 58.17% | 41.83% | 57.80% | 42.20% |
| Amphenicols | 0.00% | 100.00% |  |  |  |  |  |  |  |  |
| Beta-lactam/beta-lactamase-inhibitor | 42.43% | 57.57% | 39.23% | 60.77% | 45.98% | 54.02% | 36.04% | 63.96% | 56.39% | 43.61% |
| Beta-lactamase-inhibitors | 100.00% | 0.00% |  |  | 100.00% | 0.00% | 100.00% | 0.00% |  |  |
| First-generation-cephalosporins | 33.36% | 66.64% | 18.97% | 81.03% | 19.85% | 80.15% | 17.78% | 82.22% | 17.68% | 82.32% |
| Imidazoles | 66.29% | 33.71% | 57.08% | 42.92% | 56.81% | 43.19% | 57.26% | 42.74% | 75.43% | 24.57% |
| Lincosamides | 76.60% | 23.40% | 59.31% | 40.69% | 74.54% | 25.46% | 76.58% | 23.42% | 90.87% | 9.13% |
| Penicillins | 43.64% | 56.36% | 41.99% | 58.01% | 31.71% | 68.29% | 31.67% | 68.33% | 26.77% | 73.23% |
| Tetracyclines | 43.68% | 56.32% | 53.68% | 46.32% | 63.72% | 36.28% | 65.91% | 34.09% | 55.10% | 44.90% |
| TOTAL | **58.93%** | **41.07%** | **63.60%** | **36.40%** | **42.53%** | **57.47%** | **47.21%** | **52.79%** | **55.77%** | **44.23%** |

Note: Vietnam reported the first COVID-19 cases in January 2020 (the first wave of COVID-19 in April 2020) and transited to a 'new normal' during the COVID-19 pandemic in May 2022. Because of the potential bias due to the incomplete and non-representative nature of the dataset, the results of this table should be interpreted with caution.

Supplementary table 3. Prices of antibiotic substances (in US$) by country of origin

| Antibiotic | High income | Upper middle income | Lower middle income | Vietnam |
| --- | --- | --- | --- | --- |
| amikacin | 3.79 | 2.97 | 0.00 | 1.60 |
| amoxicillin | 0.37 | 0.00 | 0.18 | 0.17 |
| amoxicillin and beta-lactamase inhibitor | 1.14 | 3.58 | 0.65 | 0.65 |
| ampicillin and beta-lactamase inhibitor | 17.73 | 0.00 | 11.25 | 9.05 |
| azithromycin | 2.32 | 1.43 | 1.56 | 0.18 |
| cefaclor | 1.85 | 2.45 | 2.70 | 0.65 |
| cefadroxil | 0.56 | 0.00 | 1.07 | 0.38 |
| cefalexin | 0.69 | 0.25 | 0.57 | 0.29 |
| cefalotin | 17.85 | 0.00 | 11.58 | 14.36 |
| cefamandole | 20.45 | 0.00 | 0.00 | 14.60 |
| cefazolin | 3.44 | 2.99 | 2.11 | 2.17 |
| cefdinir | 3.59 | 2.53 | 1.73 | 0.78 |
| cefditoren | 2.93 | 0.00 | 2.37 | 2.28 |
| cefepime | 11.87 | 0.00 | 7.36 | 5.32 |
| cefixime | 1.58 | 0.00 | 1.44 | 0.33 |
| cefmetazole | 16.86 | 0.00 | 0.00 | 8.07 |
| cefoperazone | 10.13 | 9.74 | 0.00 | 6.39 |
| cefotaxime | 4.57 | 2.87 | 1.81 | 1.88 |
| cefotiam | 11.33 | 0.00 | 0.00 | 10.00 |
| cefoxitin | 35.30 | 9.81 | 0.00 | 14.70 |
| cefpirome | 25.70 | 0.00 | 26.01 | 12.60 |
| cefpodoxime | 2.61 | 0.00 | 0.93 | 0.71 |
| cefprozil | 6.33 | 5.34 | 3.35 | 3.21 |
| cefradine | 0.00 | 0.00 | 0.00 | 0.55 |
| ceftazidime | 6.83 | 3.36 | 3.88 | 2.83 |
| ceftazidime and beta-lactamase inhibitor | 357.45 | 0.00 | 0.00 | 0.00 |
| ceftezole | 7.64 | 0.00 | 0.00 | 5.02 |
| ceftibuten | 0.00 | 0.00 | 0.00 | 3.03 |
| ceftizoxime | 7.90 | 0.00 | 0.00 | 9.30 |
| ceftolozane and beta-lactamase inhibitor | 216.18 | 0.00 | 0.00 | 0.00 |
| ceftriaxone | 5.42 | 1.76 | 1.31 | 1.14 |
| cefuroxime | 0.95 | 0.77 | 0.52 | 0.22 |
| chloramphenicol | 0.00 | 0.00 | 0.00 | 0.78 |
| ciprofloxacin | 5.16 | 1.57 | 0.92 | 0.49 |
| clarithromycin | 1.15 | 0.00 | 2.64 | 0.16 |
| clindamycin | 9.75 | 0.00 | 0.00 | 1.18 |
| cloxacillin | 3.77 | 0.00 | 1.15 | 2.62 |
| colistin | 150.84 | 0.00 | 102.67 | 116.87 |
| daptomycin | 0.00 | 0.00 | 41.72 | 0.00 |
| doripenem | 98.92 | 86.37 | 86.83 | 88.96 |
| doxycycline | 0.07 | 0.00 | 0.02 | 0.02 |
| ertapenem | 25.88 | 0.00 | 0.00 | 22.84 |
| erythromycin | 0.97 | 0.78 | 0.00 | 0.15 |
| fosfomycin | 5.42 | 0.00 | 0.00 | 4.54 |
| fosfomycin | 37.38 | 0.00 | 21.53 | 18.93 |
| gentamicin | 0.00 | 0.00 | 0.00 | 0.17 |
| imipenem and cilastatin | 32.44 | 0.00 | 23.78 | 13.60 |
| levofloxacin | 2.91 | 4.12 | 1.08 | 0.23 |
| lincomycin | 0.00 | 0.00 | 0.00 | 0.21 |
| linezolid | 66.82 | 0.00 | 15.36 | 8.74 |
| lomefloxacin | 0.00 | 0.00 | 0.00 | 0.41 |
| meropenem | 45.65 | 33.51 | 26.91 | 12.20 |
| metronidazole | 3.76 | 2.37 | 1.40 | 1.22 |
| minocycline | 2.45 | 0.00 | 0.00 | 1.26 |
| moxifloxacin | 10.06 | 0.00 | 8.00 | 4.09 |
| netilmicin | 5.69 | 0.00 | 6.39 | 5.47 |
| norfloxacin | 0.58 | 0.00 | 0.00 | 0.06 |
| ofloxacin | 3.18 | 2.56 | 4.03 | 0.40 |
| oxacillin | 6.78 | 0.00 | 0.00 | 1.88 |
| pefloxacin | 0.00 | 0.00 | 0.00 | 0.77 |
| phenoxymethylpenicillin | 0.21 | 0.00 | 0.00 | 0.08 |
| piperacillin | 39.70 | 0.00 | 0.00 | 22.13 |
| piperacillin and beta-lactamase inhibitor | 19.12 | 0.00 | 12.50 | 17.07 |
| roxithromycin | 0.00 | 0.00 | 0.14 | 0.14 |
| streptomycin | 1.56 | 0.00 | 0.00 | 0.19 |
| sulbactam | 18.49 | 0.00 | 0.00 | 0.00 |
| sultamicillin | 2.81 | 2.43 | 0.00 | 1.65 |
| teicoplanin | 20.95 | 0.00 | 0.00 | 13.92 |
| tetracycline | 0.00 | 0.00 | 0.00 | 0.05 |
| tigecycline | 68.51 | 0.00 | 62.95 | 0.00 |
| tinidazole | 6.76 | 3.23 | 0.00 | 3.65 |
| tobramycin | 6.43 | 0.00 | 0.00 | 1.63 |
| vancomycin | 9.37 | 9.76 | 7.67 | 3.86 |
