# Supplementary figures and images for: Country of origin and prices of systemic antibiotics in Vietnam: a multicentre retrospective study
Source: JAC Antimicrob Resist. 2025 Jan 16;7(1):dlae221. doi: 10.1093/jacamr/dlae221 (PMC11735462; doi:10.1093/jacamr/dlae221)

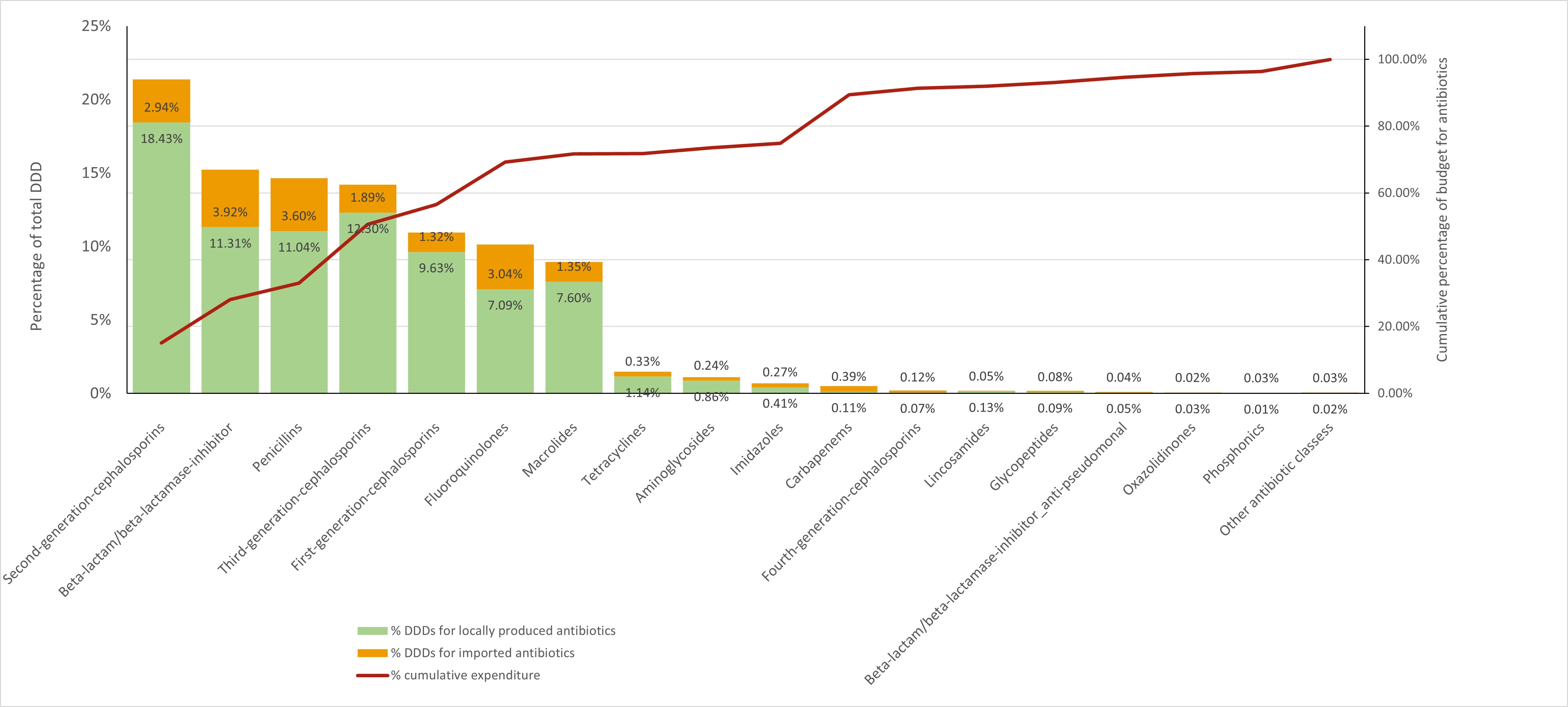

Supplement: dlae221_Supplementary_Data [file dlae221_supplementary_data.zip › Supplementary figure.jpg]
